# Composite Sequence–Structure Stability Models as Screening Tools for Identifying Vulnerable Targets for HIV Drug and Vaccine Development

**DOI:** 10.3390/v7112901

**Published:** 2015-11-04

**Authors:** Siriphan Manocheewa, John E Mittler, Ram Samudrala, James I Mullins

**Affiliations:** Department of Microbiology, University of Washington, Seattle, WA 98195-8070, USA; manocs@uw.edu (S.M.); jmittler@uw.edu (J.E.M.); ram@compbio.org (R.S.)

**Keywords:** HIV-1, capsid protein (CA), point mutation modeling, protein structural stability prediction, tolerated sequence space, destabilizing mutations, J0101

## Abstract

Rapid evolution and high sequence diversity enable Human Immunodeficiency Virus (HIV) populations to acquire mutations to escape antiretroviral drugs and host immune responses, and thus are major obstacles for the control of the pandemic. One strategy to overcome this problem is to focus drugs and vaccines on regions of the viral genome in which mutations are likely to cripple function through destabilization of viral proteins. Studies relying on sequence conservation alone have had only limited success in determining critically important regions. We tested the ability of two structure-based computational models to assign sites in the HIV-1 capsid protein (CA) that would be refractory to mutational change. The destabilizing mutations predicted by these models were rarely found in a database of 5811 HIV-1 CA coding sequences, with none being present at a frequency greater than 2%. Furthermore, 90% of variants with the low predicted stability (from a set of 184 CA variants whose replication fitness or infectivity has been studied *in vitro*) had aberrant capsid structures and reduced viral infectivity. Based on the predicted stability, we identified 45 CA sites prone to destabilizing mutations. More than half of these sites are targets of one or more known CA inhibitors. The CA regions enriched with these sites also overlap with peptides shown to induce cellular immune responses associated with lower viral loads in infected individuals. Lastly, a joint scoring metric that takes into account both sequence conservation and protein structure stability performed better at identifying deleterious mutations than sequence conservation or structure stability information alone. The computational sequence–structure stability approach proposed here might therefore be useful for identifying immutable sites in a protein for experimental validation as potential targets for drug and vaccine development.

## 1. Introduction

HIV populations are notorious for rapidly acquiring mutations that allow the virus to escape host immune responses and antiretroviral drug pressure. One proposed strategy to overcome this problem is to focus immune responses and therapies on the conserved elements of the viral proteome, regions that might reasonably be expected to have important structural and/or functional roles [1,2,3,4,5]. This strategy may also increase the likelihood that the suppressive effects induced would work against a broad array of HIV strains. Escape mutations occurring in conserved regions are also thought to be more likely to exact a fitness cost on the virus. Viral replication fitness is defined as the capacity of a virus to produce infectious progeny in a given environment [6] and is an important contributing factor in determining the prevalence of a virus variant at the population level over time [7]. High fitness costs of HIV-1 cytotoxic T lymphocytes (CTL) escape mutations have been associated with improved clinical outcomes [8,9,10,11,12,13]. However, the success of a conserved elements approach [3] depends on the ability to identify critical features of viral proteins. Recent studies have shown only a weak relationship between fitness cost and sequence conservation at mutated residues. Specifically, mutations at highly conserved sites showed varying degrees of fitness costs from deleterious to negligible [14,15,16,17]. Interestingly, mutations at sites that remain conserved through time have been shown to have greater fitness cost than mutations of amino acids that became dominant (and hence calculated to be conserved) later in the pandemic [14]. Indeed, HIV strains are continuously being imprinted by the human HLA types encountered in different human populations [18,19]. Thus, sequence conservation may change as the virus continues to evolve and adapt to host immunity, and contemporary sequence conservation may not be sufficient for pinpointing sites with crucial functional or structural roles.

Despite the rapid evolutionary rate of HIV-1, the viral capsid protein (CA) remains relatively conserved, with two-thirds of the protein having consensus amino acid frequencies of 0.9 or greater. The capsid plays several important roles in the viral replication cycle [20,21] and is an emerging target for novel HIV drugs (Reviewed in [22]). It is also a target for host cellular immune responses that have been associated with viral control in both humans [23] and in non-human primate model systems [24,25]. Recent studies suggest that specific regions of the HIV-1 Gag polyprotein, which contains the CA, remain highly conserved due to structural constraints, with sites buried in the core of the protein [26] and at CA multimer interfaces [15] being especially important. Another study reported an association between fitness costs of single amino acid changes in HIV-1 Gag and absolute changes in predicted protein stability. These findings suggest that predicted protein stability might be a useful tool for identifying amino acid residues crucial for Gag structure and function. However, only 29 of 500 residues of the Gag polyprotein were evaluated [27]. 

In this study, we utilized computational tools developed for protein structure modeling and prediction to create CA models for all possible single amino acid changes in the protein, one at a time. High-resolution three dimensional structures of HIV-1 capsid subunits, including the CA hexamer [28], the CA pentamer [29] and the hexamer of hexamer (HOH) [30], were used as templates. We then investigated whether predicted changes in CA structural stability are associated with the occurrence of mutations and the impact of these mutations on viral morphology and infectivity. Overall, we found that a majority of all possible mutations were predicted to alter CA stability, either destabilizing or hyper-stabilizing the structure. However, most of these mutations were not observed in the HIV sequence database (HIVDB) [31]. Conversely, nearly all mutations observed in the database were predicted to have negligible effects on CA protein stability. Furthermore, mutations that were predicted to change protein stability were associated with an effect on mature capsid structure and viral infectivity.

As different mutations at the same site can have different effects on the CA and viral replication [16,32], we identified potential immutable sites based on the proportion of mutations that were predicted to destabilize the protein structure. More than one-fifth of CA amino acid residues were predicted to be prone to destabilizing mutations. These vulnerable sites were all highly conserved and located in secondary structural elements throughout the protein, corresponding primarily to small clusters in the core regions and at protein-protein interfaces. About half, 26 out of 50, are also target sites of one or more HIV-1 CA inhibitors. Furthermore, multiple small regions of the CA enriched with these sites overlap with small peptides shown to induce cellular immune response and associated with viral control. In addition, we derived a composite sequence–structure stability score, which could classify deleterious and non-deleterious changes with increased accuracy.

## 2. Results

### 2.1. Initial Explorations of Different Mutation Modeling Methods and Protein Scoring Functions

Two commonly used approaches in modeling mutations in protein structures were used, fixed- and flexible-backbone modeling. For stability prediction, two protein scoring functions were applied, “Discrete optimized protein energy” (DOPE) and “FoldX energy function” (FOLDEF), against each model separately. In total then, two structural models and four predicted stabilities were obtained for each mutation. Overall, use of fixed- *vs.* flexible-backbone methods did not have a significant impact on the predicted stability of single point mutation models. There was very good agreement between predicted stabilities generated by DOPE in both amino-terminal domain (NTD) and carboxy-terminal domain (CTD) mutants (Spearman’s rho = 0.89 and 0.96, respectively; Figure S1). Lesser agreement was observed for FOLDEF stabilities (Spearman’s rho = 0.75 (NTD) and 0.68 (CTD); Figure S1), and lower correlations were observed between stability levels predicted by the two scoring functions on the same models (Figure S2).

### 2.2. Statistical- and Empirical-Based Scoring Functions Showed Different Patterns of Predicted Stabilities

For a given set of models and scoring functions, models were separated into bins based on predicted stabilities. The number of bins did not affect the overall stability distribution patterns and 20 was chosen for further analyses. All reference structures clustered into the same bin, and this bin was considered to represent the typical range of structural stability of the reference protein. Mutant models were considered to be as stable as the reference protein if they were in this bin. As the NTD and CTD models yielded similar distributions, they were combined for the purpose of presentation (separate NTD and CTD results are presented in Figures S3 and S4.

**Figure 1:**
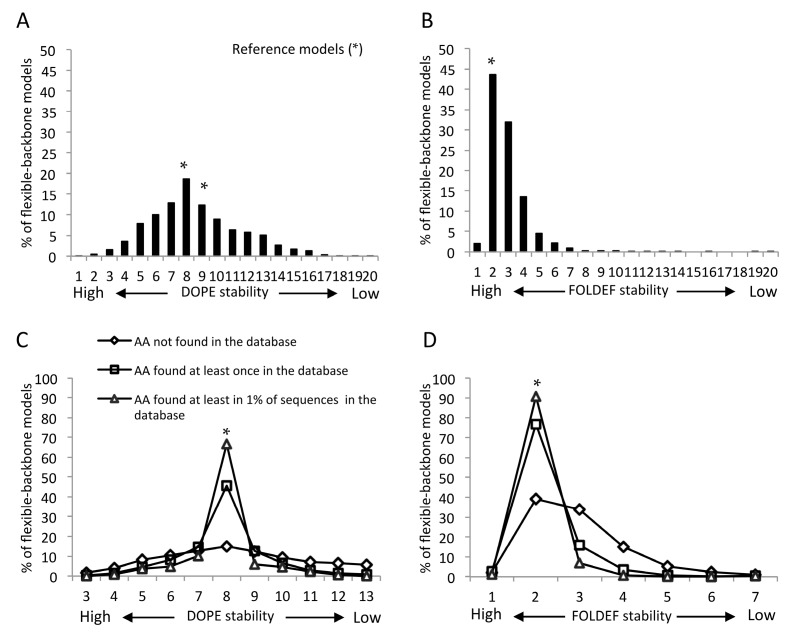
Distribution of capsid protein mutant stabilities based on flexible-backbone models of the mature capsid (CA) hexamer. The stability bin reflects the structural stability from higher (**left**) to Revise the asterisks into Palatino linotype. lower (**right**) levels. * indicates the bin in which reference structures were found. All mutations predicted by Discrete optimized protein energy (DOPE) (**A**,**C**) and FoldX energy function (FOLDEF) (**B**,**D**) were classified into three groups based on their frequency in the HIV sequence database. Only results from five higher, five lower and the reference model bins are shown, as together they accounted for more than 98% of all models.

Using the DOPE scoring function, the predicted stabilities of mutant models had a normal distribution, with the peak being the same bin representing the structural stability of the reference protein. About one-fifth of the flexible-backbone models were predicted to be as stable as the reference structures and roughly equal numbers of the remaining mutants were predicted to be more or less stable (Figure 1A and Figure S3). In contrast, using the FOLDEF scoring function, almost half of the mutant models were predicted to be as stable as the reference models. The other half were predicted to have lower stability and only ~2% were predicted to be more stable (Figure 1B). Similar predicted stability distributions were observed using fixed-backbone modes, notwithstanding a larger variation in FOLDEF stabilities for the reference models (Figure S3).

### 2.3. Influence of Input Templates on Predicted Stabilities

HIV-1 capsid exists in two different stages—immature and mature. The mature capsid is made up of two different types of CA subunits, hexamers and pentamers, while only hexameric lattices have been identified in the immature capsid [33]. Using the mature hexamer or pentamer as template yielded highly correlated changes in stabilities (Pearson’s *r* = 0.9; Figure S5A). As expected, we observed much greater divergence in the predicted stabilities between the immature and mature hexamers (Figure S5B). The CA–CA interactions observed in the mature hexamer and pentamer were comparable [28,29], while the arrangement of CA in immature HIV-1 is different from that of the mature capsid [28,29,33].

For the carboxy-terminal domain (CTD) mutations, the predicted change in the stability based on the CTD dimer [34] and the CTD of the hexamer of hexamer (HOH) [30] were moderately correlated (Pearson’s *r* = 0.73; Figure S5C). The largest discrepancies were observed for mutations at residues surrounding the dimerization and the trimerization interfaces (residues 175, 177, 178, 188, 201, 204, 207 and 208; Figure S5D).

### 2.4. Mutants Observed in the HIVDB Were Predicted to Have Stabilities Close to the Reference Models by Both Scoring Functions

When considering all mutations, the mutant models had a large range of predicted stabilities (Figure 1, Figure S3 and Figure 5). However, when focusing on mutations that had been observed in the HIV database, the range of predicted stabilities decreased sharply, with the majority of the observed mutants clustered in the same stability bin as the reference model. This pattern was most pronounced for mutations observed in at least 1% of all sequences in the database. In contrast, the predicted stabilities of mutations that have not been observed in the database were widely distributed (Figure 1C,D). These predicted stability patterns occurred with all prediction sets, irrespective of the modeling methods, scoring functions or templates (Figure S4).

### 2.5. Frequency Threshold of Tolerated Mutations

**Figure 2:**
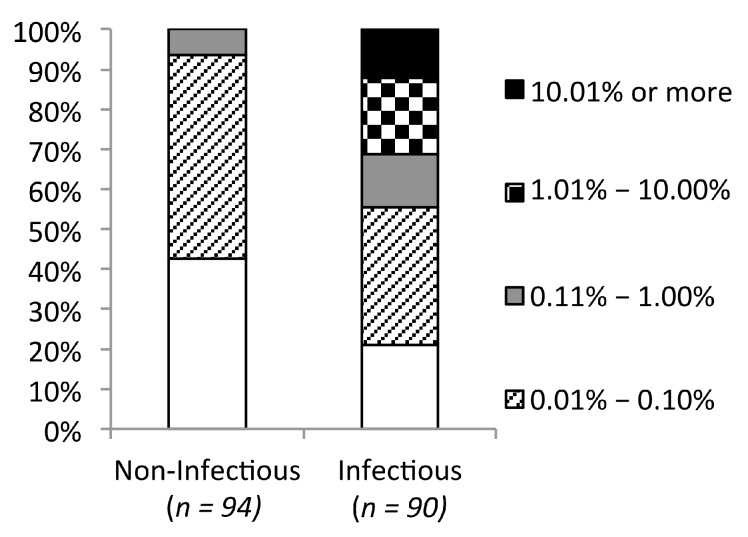
Mutations observed in a database of 5811 HIV-1 capsid sequences. Mutations resulting in non-infectious or infectious viruses are shown separately and stratified based on their database frequencies. White boxes represent the percentage of mutations not found in the database.

The observation that the majority of observed mutations were not predicted to alter protein stability, in contrast to unobserved mutations, suggests that any mutation found in the database has a high likelihood of being tolerated. To explore this question, we examined the frequency of 184 point mutations with known impact on infectivity in subtype B HIV-1. The mutated residues were scattered throughout the CA, with 70% located in the NTD and the rest in the CTD. Seventy percent of the mutations were random, the other approximately 20% were alanine substitutions and the rest were the most frequently observed mutations [9,11,14,16,35,36,37,38,39]. As the previous studies did not always use the same experimental methods to determine viral infectivity, we used our own threshold for separating infectious and non-infectious mutations. The mutant viruses were considered non-infectious when no viral production was observed or when the reported infectivity was lower than 1% of the wild type virus. Ninety-four (48%) of these mutations resulted in non-infectious viruses defined in this way. Forty of the 94 (42.5%) mutations that destroyed infectivity were not found in the database, while the remainder were found at least once. However, none were found in more than 0.2% (11 of 5811) of the sequences. While the database frequency of inactivating mutations ranged from 0% to 0.2%, the frequency of infectivity-conserving mutations ranged from 0% to 36%. Nineteen out of 90 of the latter mutations (~21%) had not been observed in the HIVDB, while 37 (41%) were present in more than 11 sequences (Figure 2). Overall, inactivating mutations appeared at a significantly lower frequency than tolerated mutations (*p* = 3.03 × 10^−8^; Mann–Whitney *U* test). Using mutation frequency as a predictor for the impact on mutant infectivity, the threshold of 0.2% yielded the best prediction accuracy (64%) (Table 1).

**Table 1:** Accuracy of using mutation frequency or change in structural stability to predict viral infectivity in binary classification manner.

Predictor	Sensitivity ^a^	Specificity ^b^	Precision ^c^	Accuracy ^d^
Mutation Frequency ^e^	59.5%	70.0%	67.10%	64.10%
Stability of Reference Models ^f^	73.33%	78.72%	76.74%	76.12%
Composite Score ^g^	80.00%	79.57%	78.16%	79.78%

^a^ Sensitivity = (True positive)/(True positive + False negative); ^b^ Specificity = (True negative)/(True negative + False positive); ^c^ Precision = (True positive)/(True positive + False positive); ^d^ Accuracy = (True positive + True negative)/(True positive + True negative + False positive + False negative); ^e^ Mutations with a database frequency of 0.2% or less are predicted to result in non-infectious virus; ^f^ Mutants with structural stability higher than the reference models are predicted to result in non-infectious virus; ^g^ Mutants with a composite score (sum of ranks of frequency and FOLDEF stability) higher than 175 are predicted to result in non-infectious virus.

### 2.6. Genetic Barrier Influences the Emergence but Not Outcomes of Amino Acid Changes

**Figure 3:**
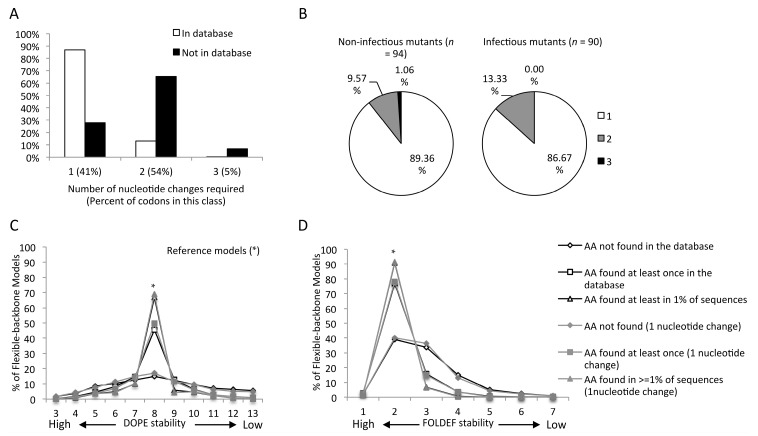
Genetic barrier influences the emergence of amino acid mutations but not impact of mutations on viral infectivity: (**A**) Number of nucleotide changes required for mutations observed and not observed in the HIV database of 5811 sequences; (**B**) Number of nucleotide changes required for mutations whose impact on infectivity was tested; amino acid mutations classified into three groups based on their frequency in the HIV database with stability predicted by DOPE (**C**) and FOLDEF (**D**) using the mature CA hexamer as the template.

A considerable proportion of amino acid substitutions have not been observed despite having minimal impact on protein stability (Figure 1C,D and Figure S4). Fifty-nine percent of all possible amino acid substitutions from the consensus sequence required two or more nucleotide changes. However, this proportion increased to 72% for undetected mutations and decreased to 13% for observed mutations (Figure 3A), indicating that a genetic barrier plays a role in emergence of amino acid changes in the CA. However, substitutions requiring single nucleotide changes were not further enriched when considering mutations observed in 1% or more of the sequences, although there was an increase in the proportion of transition mutations compared to transversion mutations (Figure S6A). There was no association between genetic barrier, *i.e.*, amino acid changes requiring an increased numbers of base changes, and effect of mutations on viral infectivity or protein stability (Figure 3B–D and Figure S6B).

### 2.7. Predicted Stabilities in Relation to the Impact of Mutations on Mature Capsid Morphology and Viral Infectivity

**Figure 4:**
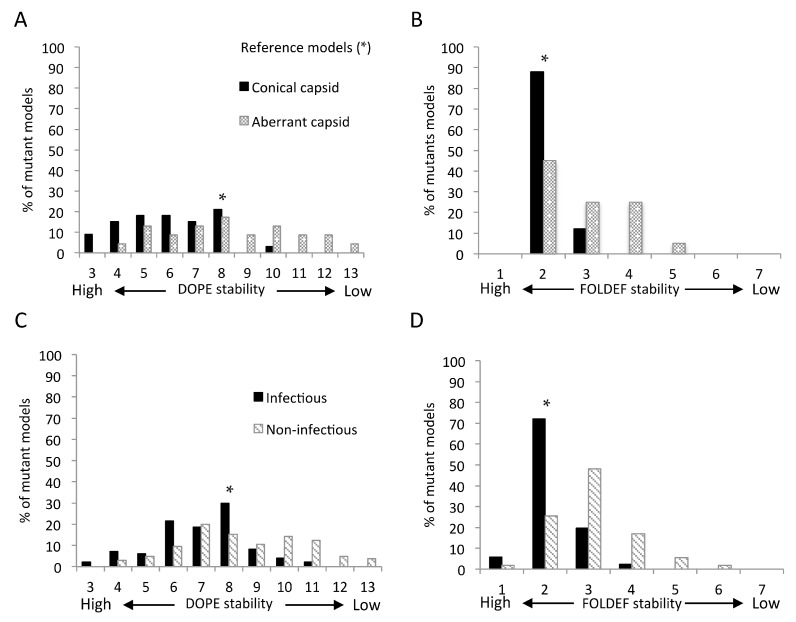
Predicted stabilities of mutations with known phenotypes. Flexible-backbone models were predicted by DOPE (**A**,**C**) and FOLDEF (**B**,**D**) and compared to capsid structure (**A**,**B**) and virus infectivity (**C**,**D**).

The low percentage of observed mutations with different stabilities from the reference models hints at optimal protein stability being crucial for CA function. Two datasets were used to explore whether predicted stability was also predictive of capsid structure and virus infectivity. The first consisted of 56 single amino acid substitutions with known mature capsid morphology [35,36,37,38]. Twenty-three were reported to result in aberrant capsid shape, while the other 33 had no obvious impact on capsid morphology. Using flexible backbone models of the mature CA hexamer and CTD dimer, 88% of mutations resulting in a native conical shape were predicted to be as stable as the reference by FOLDEF. With DOPE, a majority of mutations resulting in a conical capsid shape were predicted to be more stable than the reference models, 76% (*vs.* 21% predicted to be as stable) (Figure 4A,B). Similar results were obtained using fixed-backbone models of the same template structures (data not shown).

**Figure 5:**
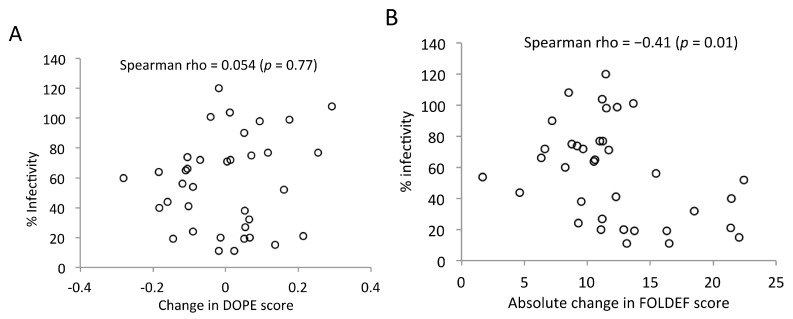
Relationship between viral infectivity and change in stability compared to the reference structure. As predicted by (**A**) DOPE and (**B**) FOLDEF. Each open circle represents a point mutation (viral infectivity data taken from [16]).

**Figure 6:**
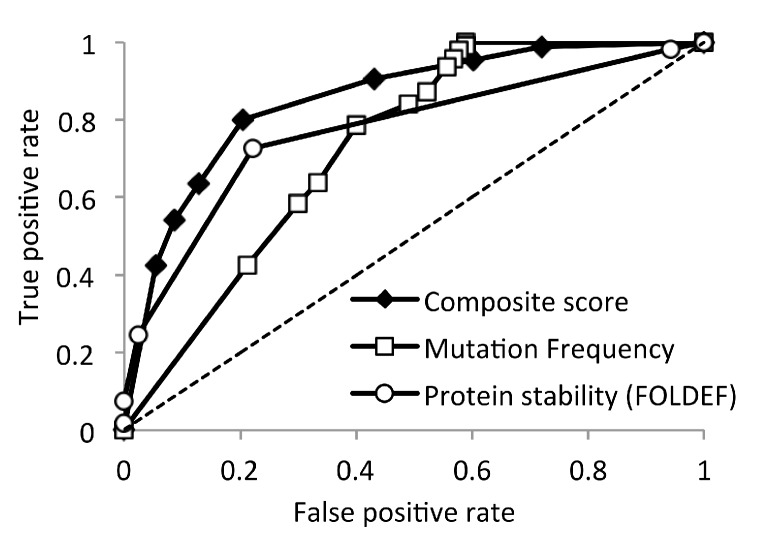
Receiver operating characteristic curve of HIV-1 subtype B non-infectious mutations predictions. The composite score is the sum of the FOLDEF-stability rank and the mutation-frequency rank.

The second dataset included an additional 128 substitutions with known impact on infectivity in a subtype B virus backbone [9,11,14,16,39]. The stability distributions of infectious and non-infectious mutations resembled those of conical capsid and aberrant capsid conferring mutations (Figure 4). Using FOLDEF with the mature CA hexamer and CTD dimer, mutants predicted to be as stable as the reference structures were associated with both conical capsid shape (*p* = 5.4 × 10^−4^; Fisher’s exact test) and infectiousness (*p* = 2.76 × 10^−10^). We also observed a moderate correlation between absolute changes in FOLDEF, but not DOPE, stability and viral infectivity (Figure 5). Using the stability level of the reference structures as the threshold, any mutation resulting in a less stable structure was predicted to be non-infectious, with a prediction accuracy of 75% when the CA hexamer and the CTD dimer were used as the template structure, respectively. The accuracy improved slightly to 76% when the CTD of the HOH were used as the template structure for CTD mutations. This combination of template structures yielded the best prediction accuracy (Table S1). Compared to mutation frequency, protein structural stability, as predicted by FOLDEF, performed better in classifying infectious *vs.* non-infectious mutations (Table 1 and Figure 6).

### 2.8. More than One-Fifth of HIV-1 CA Is Prone to Destabilizing Mutations

**Figure 7:**
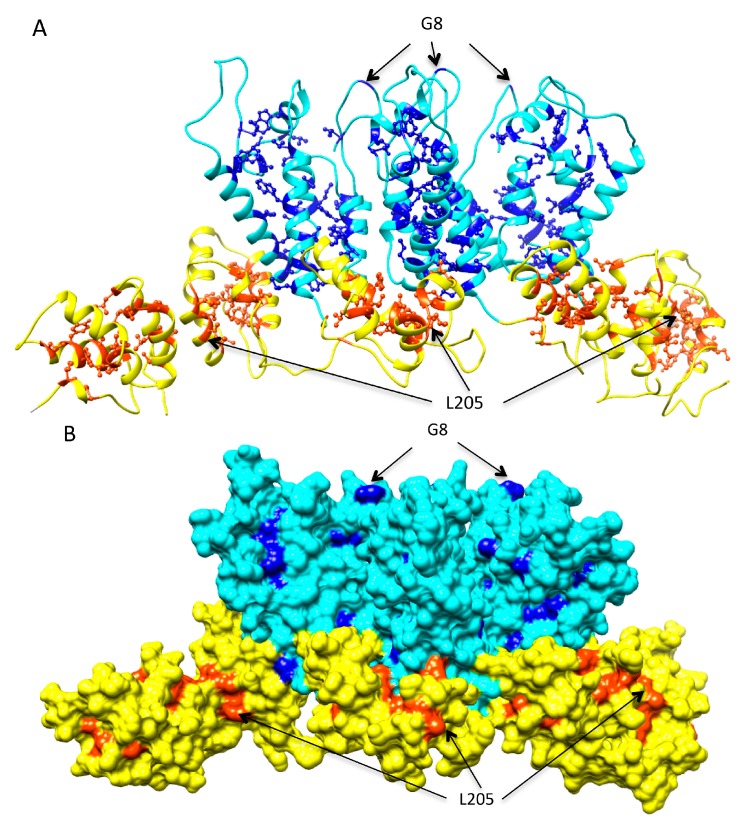

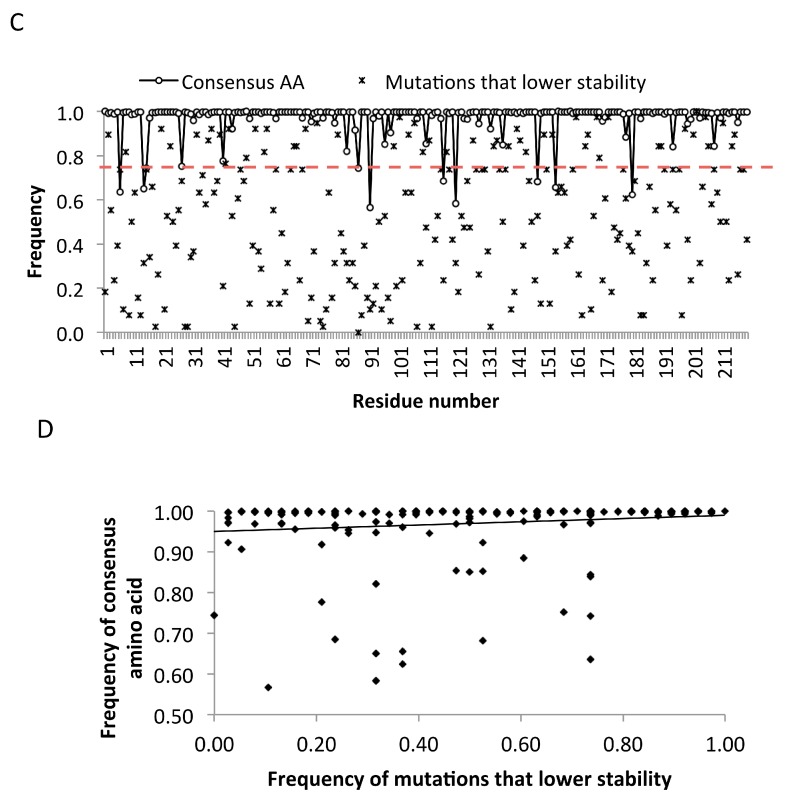
HIV-1 capsid (CA) sites prone to destabilizing mutations. (**A**,**B**) The amino-terminal domain (NTD) is shown in cyan and the carboxy-terminal domain (CTDd) is in yellow with the sites prone to destabilizing mutations highlighted in blue and orange; (**A**,**B**) side-view of three CA chains and two additional CTD from a hexamer of the hexamer of hexamer (HOH); (**B**) showing the solvent accessible surfaces; and (**C**) the database frequency of the consensus amino acid (open circle) and the frequency of mutations that lower stability (“×”) at each CA residue. The dash line shows the frequency of 0.75. (**D**) There is no linear relationship between sequence conservation and the frequency of mutations that lower stability. Each dot represents a mutation.

As non-infectious mutants were more likely to be associated with lower stability, we speculated that a large proportion of destabilizing mutations at a site suggests low mutational tolerance. We identified 50 residues in the CA that were prone to destabilizing mutations (Figure 7 and Table S2). At least three-fourths of all possible mutations at these sites were predicted to lower protein stability by both scoring functions using the mature CA hexamer and the CTD of the HOH as templates. Most of these residues were located in secondary structure elements of the protein with small solvent accessible surface areas—many had side-chains almost completely buried. Exceptions were G8, located in the β-hairpin of the NTD, and L205, located in helix 10. These residues were either situated in the core of the CA, or at the CA intra-hexamer or inter-hexamer interfaces (Figure 7A,B and Table S2). These regions were shown to be genetically fragile, with low tolerance for amino acid substitutions [15,16,30]. All fifty residues were extremely highly conserved, with consensus amino acid frequencies ranging between 0.98 and 0.99. However, we did not observe a linear relationship between sequence conservation and the frequency of mutations that lower the stability (Pearson’s correlation *r* = 0.15, Figure 7D).

### 2.9. Clusters of Sites Prone to Destabilizing Mutations Significantly Overlap with Peptides Shown to Induce Immune Response and Associated with Viral Control

**Figure 8:**
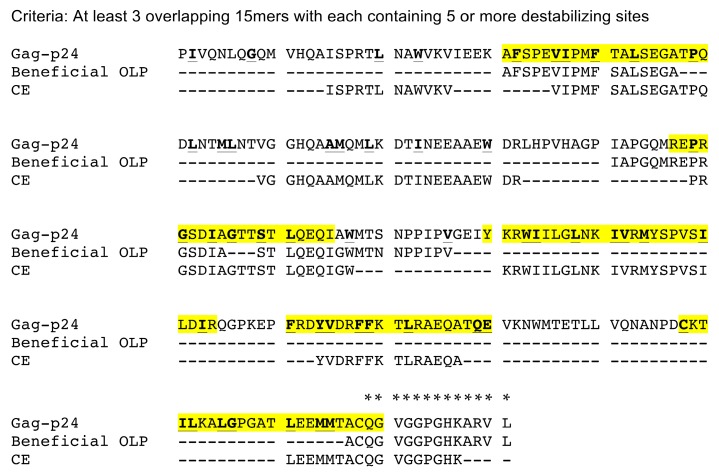
Clusters of sites prone to destabilizing mutations overlap with peptides shown to induce robust immune response and be associated with viral control. Positions prone to destabilizing mutations are underlined and bolded in HIV-1 Gag-p24 subtype B consensus sequence. Yellow regions depict four continuous stretches of sites found in three or more overlapping 15 amino acid windows with each window containing five or more destabilizing sites. CE peptides [3,14,41] and “Beneficial” peptides from [42] were previously shown to induce robust immune responses [43,44] and associated with viral control [42], respectively. * denote residues not included in all the template structures examined and, hence, excluded from the analysis.

To identify candidate regions for CTL vaccine immunogens design, we searched the CA by assessing sliding windows of 15 amino acids, one amino acid at a time, and counted the number of sites prone to destabilizing mutations within each window. The window of size 15 was selected to cover the length of known CTL epitopes [40]. We detected four linear regions in the CA that are enriched with sites prone to destabilizing mutations (Figure 8). These candidate regions were defined as a continuous stretch of sites found in three or more overlapping windows with each window having five or more destabilizing sites. These clusters of sites prone to destabilizing mutations were significantly associated with both “conserved elements” (CE) peptides [3,14,41] and ”beneficial” CA peptides [42] (*p* < 0.0001; Chi-square and Fisher’s exact test). As shown in Figure 8, all four regions overlap with four of seven previously identified CE peptides, which were shown to induce robust cellular and broad humoral immune responses in macaques [43,44]. Interestingly, the four regions also overlap with three CA peptides associated with viral control [42] (Figure 8). One of the CE peptides and one of the beneficial peptides are located at the carboxy-terminal end of the CA. This region is missing in the template protein structures and, hence, was excluded from our analyses (Figure 8).

### 2.10. Composite Sequence–Structure Score for Predicting the Impact of Mutations

Low database frequency and low stability were individually associated with deleterious mutations. Conceivably, mutations with both undesirable properties would be more likely to be lethal than mutations with only zero or one of these properties. We therefore derived a composite sequence–structure score based on the ranking of database frequency and predicted structural stability. Mutations were ranked from low to high based on the degree of undesirable properties, *i.e.*, mutations with the lowest database frequency and lowest stability were assigned the lowest rank. This simple composite score predicted which viruses would be infectious or non-infectious more accurately than either mutation frequency or structural stability alone (Table 1 and Figure 6).

## 3. Discussion

Identifying and targeting critical elements of the HIV proteome remains a challenging task for therapy and vaccine development. Sequence conservation is often considered a proxy for functional or structural importance of a given amino acid site. However, only weak relationships between sequence conservation and fitness cost of mutations in the HIV-1 CA have been reported [14,15,16,27]. In one study, a stronger association was found between the predicted changes in protein stability and the fitness cost of a mutation [27]. In this study, we expanded the analysis to a much larger set of HIV-1 CA mutations with two known phenotypes: capsid morphology and infectivity, to more accurately assess the ability of protein structure-stability models to predict the impact of mutations. We also analyzed results using two different protein structure-stability modeling approaches. Our results support the previous observation that *in silico* structural stability can be applied to identify potentially inactivating mutations in HIV-1 proteins [27,45,46].

To reaffirm the result from previous sequence conservation studies, we analyzed the database frequency of 184 HIV-1 CA mutations with reported phenotypes. It should be noted that the reported phenotypes of these 184 point mutations depend upon the experimental assay and criteria used, which varied across studies. In an attempt to reconcile differences, we applied the same criteria across all studies to reclassify mutations as deleterious or non-deleterious. In some of these studies, only particular types of mutations, alanine or the most frequently observed substitutions, or mutations within specific regions of the CA were investigated. However, the largest dataset, accounting for more than sixty percent of the 184 mutations, were randomly generated. After combining all data, there was no strong bias in the type of mutation or the structural locations of the mutated residues. We found that none of the non-infectious mutations appeared in more than 0.2% of the CA sequences in the database, while the database frequency of infectiousness retaining substitutions varied much more widely (Figure 2). This observation suggests that database frequency is still partially informative of the mutation outcome and that the frequency threshold for tolerated mutations is likely to be lower than 1%, a level presumed in previous studies in other HIV-1 proteins [45,46]. Using a mutation frequency of 0.02% as the cutoff value, infectious and non-infectious mutations could be classified with 64% accuracy, which is still inferior to the 76% and 80% accuracy achieved by the structural stability and composite score approaches, respectively (Table 1). The threshold mutation frequency is likely to fluctuate over time and across different viral populations, as the virus continues to evolve to escape host immune pressures and these mutations become fixed in circulating viruses. Antiviral treatment imposed selective pressures are also being introduced as evidenced by the increasing prevalence of drug resistant mutations, which were rarely observed before antiretroviral therapy become widely available [47]. Some of these mutations are being fixed along with compensatory mutations if the immune/drug escape mutations reduce viral fitness [11,48,49,50]. This may explain, as noted previously, why amino acids conserved through time are better indicators of necessary function than mutations that have emerged as consensus more recently [14]. It remains challenging to determine what threshold value would be most effective for predicting deleterious mutations.

In this study, mutations predicted to induce large alterations in the structural stability of the CA hexamer and the inter-hexamer interactions were far less likely to be found in the HIV sequence database than those not predicted to alter stability. Among the set of 184 HIV-1 CA variants that had been characterized *in vitro*, all viruses with very low predicted stability had aberrant capsids and were non-viable (Figure 4). These results support the idea that drugs or vaccines that target regions of the capsid in which putative escape mutants would reduce stability would be particularly effective. The optimal structural stabilities required of HIV-1 CA monomer and multimers to retain proper function are likely to be maintained in the same host cellular environment, regardless of extracellular immune or treatment selective pressures. All sites predicted to be prone to destabilizing mutations were highly conserved (Table S2). These sites were located in the core of individual HIV-1 CA or at intra-hexamer and inter-hexamer interfaces. More than half have also been identified as the binding sites of at least one CA inhibitor (Table S2). Multiple drug binding pockets have been identified in CA hexamer and CTD dimer [22]. Natural polymorphisms at drug binding sites as well as *de novo* drug resistant mutations can compromise HIV susceptibility [51,52,53] Nevertheless, the escape mutations could incur fitness costs and require multiple compensatory mutations, as shown in [54]. The analyses presented here can facilitate selection of potential target sites with least tolerance for mutations for further drug development. 

Several observational studies, including *post-hoc* analyses of HIV CTL vaccine clinical trials [55], showed an association between HIV control and CD8+ T-cell preferential recognition of Gag [23,56,57]. Additional studies revealed that only a subset of recognized CTL epitopes could be linked to clinically beneficial outcome [42,58]. Though the exact molecular mechanisms underlying the protective effects of these beneficial epitope specific CTL responses remain unclear, the fitness cost of CTL escape mutations has been identified as an important contributing factor [59,60]. In this study, we identified four regions of the CA that contains clusters of sites predicted to be prone of destabilizing mutations. These regions largely overlap with four of the CE peptides [3,14,41] shown to be capable of inducing multifunctional cellular immune responses in non-human primates when separated from the full-length Gag protein, that latter of which results in immunodominant responses to other regions of the protein [43,44]. They also overlap with the beneficial peptides associated with viral control in the large cohort of infected patients [42] (Figure 8). A recent study by Hancock, *et al.* [58] showed a strong association between CD8+ T cell mediated viral inhibitory activity and the magnitude of T cell responses to these beneficial peptides. These observations support the utility of our structural-stability based approach in identifying vulnerable regions of HIV proteome for the rationale design of vaccine immunogens.

The structural stability based approach has disadvantages. It does not directly address stabilizing yet deleterious mutations. About twenty percent of debilitating mutations did not alter CA hexamer or CTD dimer stability. Additionally, about 20% of undetected mutations also had a minimal impact on protein stability. While it is likely that the stability prediction was not entirely accurate, it is also probable that some mutations confer deleterious effects by disrupting other molecular processes, such as CA interactions with other proteins or protein expression and processing [16,35], or hydrogen bonding [15], without significantly altering protein structural stability. In addition, we hypothesize that some portion of these undetected mutations may not have been observed due to a higher genetic barrier, as we found that amino acid changes requiring a lower number of nucleotide changes were highly associated with the emergence of amino acid substitutions. The bias toward mutations with low genetic barrier may actually incorporate a bias toward more conservative amino acid changes, which are generally believed to induce smaller changes to protein structure and function. Despite this expectation, we did not find any association between the level of genetic barrier and the deleterious effects of mutations (Figure 3B–D).

The performance of the structure-stability approach depends on the accuracy of the protein modeling and stability prediction methods. We examined the dependency of the predicted stability of mutant structures on the computational methods used. We found that using different scoring functions resulted in greater variation in the predicted stability of single point mutations than using different modeling methods. Our results support FOLDEF as a more suitable scoring function than DOPE for predicting the impact of mutations. FOLDEF performed better at classifying deleterious and non-deleterious mutations, and the absolute change in FOLDEF stability negatively correlated with changes in viral infectivity, coinciding with results from a previous study [27]. However, further studies are necessary to assess the performance consistency of these scoring functions across different proteins. Using FOLDEF, we achieved 76% accuracy in predicting deleterious mutations (Table 1).

In addition to the scoring function, we found that template structures used for *in silico* mutations could also affect predicted stability and, hence, predictions of deleterious mutations. The HIV-1 capsid exists in two different stages with distinct morphologies. While both the mature and immature capsids consist mostly of CA hexameric lattices, CA conformations and CA-CA interactions found within and between these CA hexamers vary [30,33]. The immature and mature CA hexamer mutant models gave moderately correlated predicted stabilities (Pearson’s *r* = 0.53, Figure S5B). In comparison, different subunits of the mature capsid produced better correlated predicted stabilities, especially between the hexamer [28] the pentamer [29] (Pearson’s *r* = 0.90, Figure S5A). A slightly larger discrepancy was observed between the CTD dimer [34] and the CTD of the HOH [30] (Pearson’s *r* = 0.73, Figure S5C). The largest differences were at the residues located in the CTD trimerization interfaces found in the HOH but not in the CTD dimer. Exclusion of these eight residues improved the correlation from 0.73 to 0.83 (Figure S5D). Interestingly, the stability patterns of observed and unobserved mutations were similar across all template structures (Figure S4). With the dataset of 184 CA mutations, the stability of the mature capsid structure was a better predictor of deleterious mutations than the immature structure (Table S1, Figure 4, and Figure S7). The stability prediction of the immature structure may suffer from the lack of CA-CTD-SP1 (spacer peptide 1) region, which was shown to be critical for the immature capsid assembly [33,61,62]. These results showed the sensitivity of the stability prediction to the available structural information and, hence, the interpretation of results must carefully consider the biological properties or roles of the template structure.

As computational power and techniques for protein structure and stability predictions continue to improve, the performance of our approach could likewise be improved. It should be noted that our *in silico* mutation step did not include a molecular dynamic (MD) step, however, a previous study [63] observed no relationship between MD structural convergence and the FOLDEF stability. Using any one MD snapshot as the input template yielded poorer predicted stability, while using the MD-averaged structure gave comparable results to the initial crystal structure [63]. There are several approaches for MD simulations and different methods may improve the stability prediction. However, given the resources needed for detailed MD analyses with a large number of mutant models, we did not explore them in this study. To the best of our knowledge, there is not yet an effective way to computationally predict point mutations that drastically alter the protein tertiary structure without affecting protein stability. Our current method does not account for this possible, but rare, effect of mutations.

As sequence conservation alone has shown limited success in determining outcome of mutations and the structural-stability based approach has its own limitations, we explored the use of both types of information and found that a composite score performed better at classifying deleterious and non-deleterious mutations than either approach considered alone. The combined sequence–structure approach described here has the potential to serve as a target-screening tool for HIV drug and vaccine development. In this study, only single amino acid changes were studied, whereas compensatory mutations can arise during viral infection that can restore protein stability and function [64,65,66]. In addition, all sequence, structure and experimental data used in our analyses were obtained using HIV-1 subtype B viruses. The impact of mutations may differ in other genetic backgrounds. The *in silico* mutation and stability predictions proposed here can readily be used to study multiple amino acid changes in different background sequences. The CA input structure for different HIV subtypes can be generated using homology-modeling techniques before introduction of additional mutations. However, using the model as the template, as well as introducing multiple mutations, may further reduce stability prediction accuracy [45,46,63]. In addition, generating all possible combinations, even for double mutations, is still very computationally expensive. More importantly, experimental data on multiple CA mutations, or single mutations in other HIV subtypes, are very limited or non-existent. The lack of experimental data hinders these crucial additional analyses. Thus, further studies of the effect of more complicated mutational patterns on protein stability in multiple genetic backgrounds will provide further insight for identifying desirable targets for HIV vaccines and therapies.

## 4. Materials and Methods

### 4.1. In Silico Mutagenesis

All 19 possible amino acid changes were introduced *in silico* at each position in the HIV-1 CA protein, one at a time. Reference structures in which the starting amino acid was re-introduced into the structure were also generated and used as a control set. The mature CA hexamer, Protein data bank identification number (PDB ID) 3H4E [28] and pentamer, PDB ID 3PO5 [29] were used as template structures for mutations in the amino-terminal domain (NTD; residue 1 to 147). While the known CA hexamer and pentamer structures also include CTD, they do not contain the CTD dimerization and trimerization interface, shown to be crucial for mature capsid structure and function [30,32,35]. Hence, the CTD dimer, PDB ID 1A43 [34] and the hexamer of hexamer (HOH), PDB ID 3J34 [30], were used as template structures for mutations in the carboxy-terminal domain (CTD; residue 148 to 219, including the linker region). The immature CA hexamer, PDB ID 4USN [33], was used for both NTD and CTD mutations.

Two *in silico* mutation modeling approaches were applied: Fixed-backbone models explore the best-fit side-chain conformation while the main chain atoms of the mutated residue are kept unchanged from the original position in the template structure. The side-chain atoms of the mutated residue were replaced with those of the new amino acid. The best-fit side chain conformation was selected using the SCWRL program version 4.0 (Fox Chase Cancer Center, Philadelphia, PA, USA) [67]. The model was then run through 200 steps of energy minimization to remove atomic clashes or internal constraints generated by side-chain replacement using the CHARMM force field, as implemented in the program NAMD version 2.8 (University of Illinois, Champaign, IL, USA) [68]. Flexible-backbone models allow the main chain atoms to move along with the side chains and the best-fit combination is selected. These were generated in the FOLDX program suite [69] using the BUILDMODEL function with default parameters. This method allows the neighboring side-chains to be moved in order to explore alternative backbone conformations.

### 4.2. Proteins Stabilities

Two types of protein-scoring functions were used to assess mutant models: The atomic distance-dependent statistical based scoring function referred to as Discrete Optimized Protein Energy (DOPE) [70] and the empirical based force field energy function called FOLD-X energy function (FOLDEF) [71]. DOPE is part of the protein-modeling package MODELLER [72]. FOLDEF is part of the FOLDX program suite [69]. Both programs were run using default parameters.

### 4.3. Sequence Dataset and Amino acid Database Frequencies

Full-length HIV-1 subtype B CA coding sequences were downloaded from the HIV database [31]. Any sequences with hypermutations [73], early stop codons, frame-shift mutations or ambiguous amino acids were excluded. This resulted in a dataset of 5811 subtype B sequences. A multiple sequence alignment was prepared using MUSCLE [74] and then manually edited using Mesquite [75]. The database frequency of each amino acid at each site in the final alignment was then calculated using a perl script [76]. 

### 4.4. Composite Sequence–Dtructure Stability Score

A composite score was derived from the mutation database frequency and FOLDEF stability of the mutant flexible backbone model of the mature CA hexamer and CTD dimer for NTD and CTD mutations, respectively. First, all mutations were ranked by database frequency in ascending order. Mutations with the lowest database frequency, *i.e.*, 0%, were given the lowest frequency rank. Next, all mutations were ranked by FOLDEF stability in ascending order, *i.e.*, mutant models with the lowest stability were given the lowest stability rank. For each mutation, the two ranks were added to get a composite score.

## Supplementary Materials

Supplementary materials can be found at http://www.mdpi.com/1999-4915/7/11/2901/s1.
