# Supplementary material for: Composite Sequence–Structure Stability Models as Screening Tools for Identifying Vulnerable Targets for HIV Drug and Vaccine Development
Source: Viruses. 2015 Nov 4;7(11):5718–35. doi: 10.3390/v7112901 (PMC4664974; doi:10.3390/v7112901)
Supplement: Supplementary file 1 [file viruses-07-02901-s001.pdf]

viruses

Supplementary Information

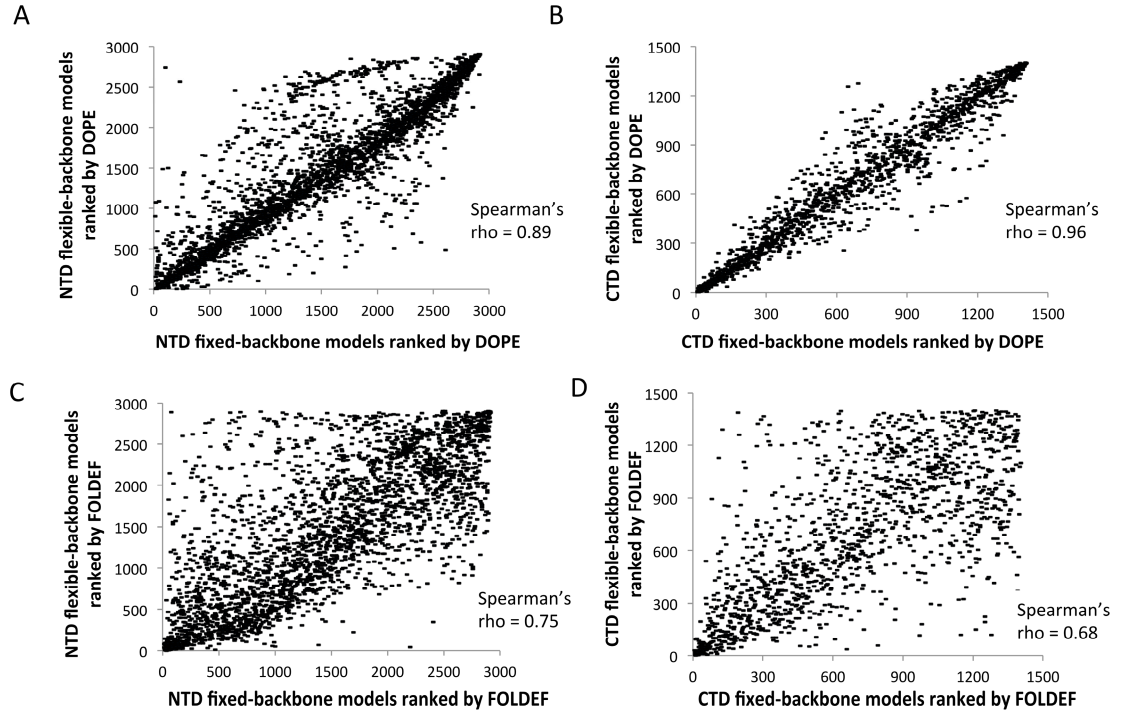

Figure S1. Fixed- and flexible-backbone models have highly correlated predicted stabilities. Each dash represents a model. The models were ranked based on stability from the lowest to the highest. Rank correlation of NTD and CTD mutants as predicted by DOPE (**A**, **B**), and FOLDEF (**C**, **D**).

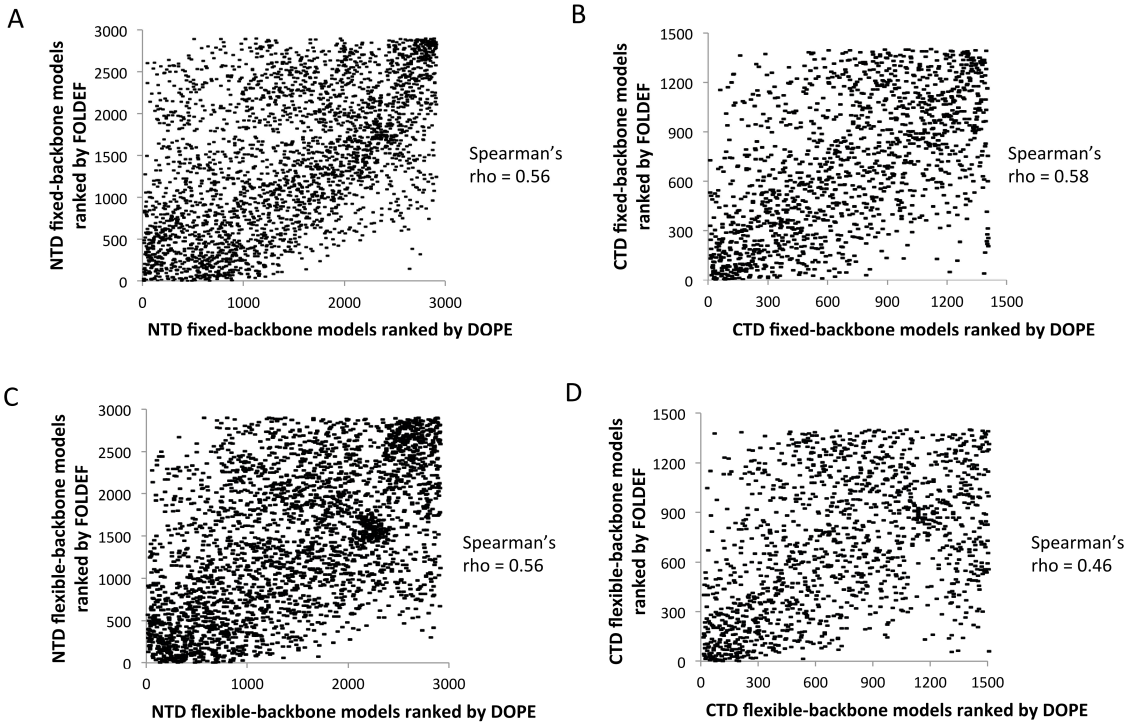

Figure S2. Rank correlation between DOPE and FOLDEF stabilities. Each dash represents a model. The models were ranked based on stability from the lowest to the highest. Rank correlation of NTD and CTD using a fixed- (**A**, **B**) or flexible-backbone model (**C**, **D**).

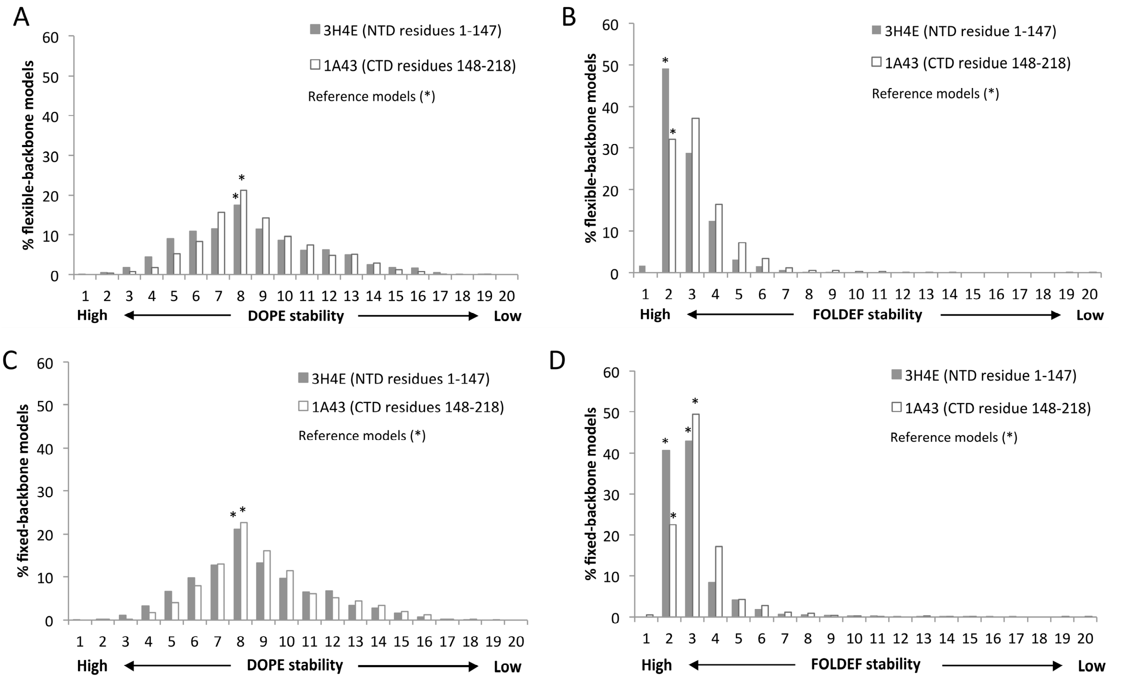

Figure S3. Distribution of the NTD and CTD mutant stabilities. (A, B) Stabilities of flexible-backbone models as predicted by (A) DOPE and (B) FOLDEF; (C, D) Stabilities of fixed-backbone models as predicted by (C) DOPE and (D) FOLDEF. The score bin reflects the structural stability from higher (negative) to lower (positive) level. * indicates stability of the reference structures.

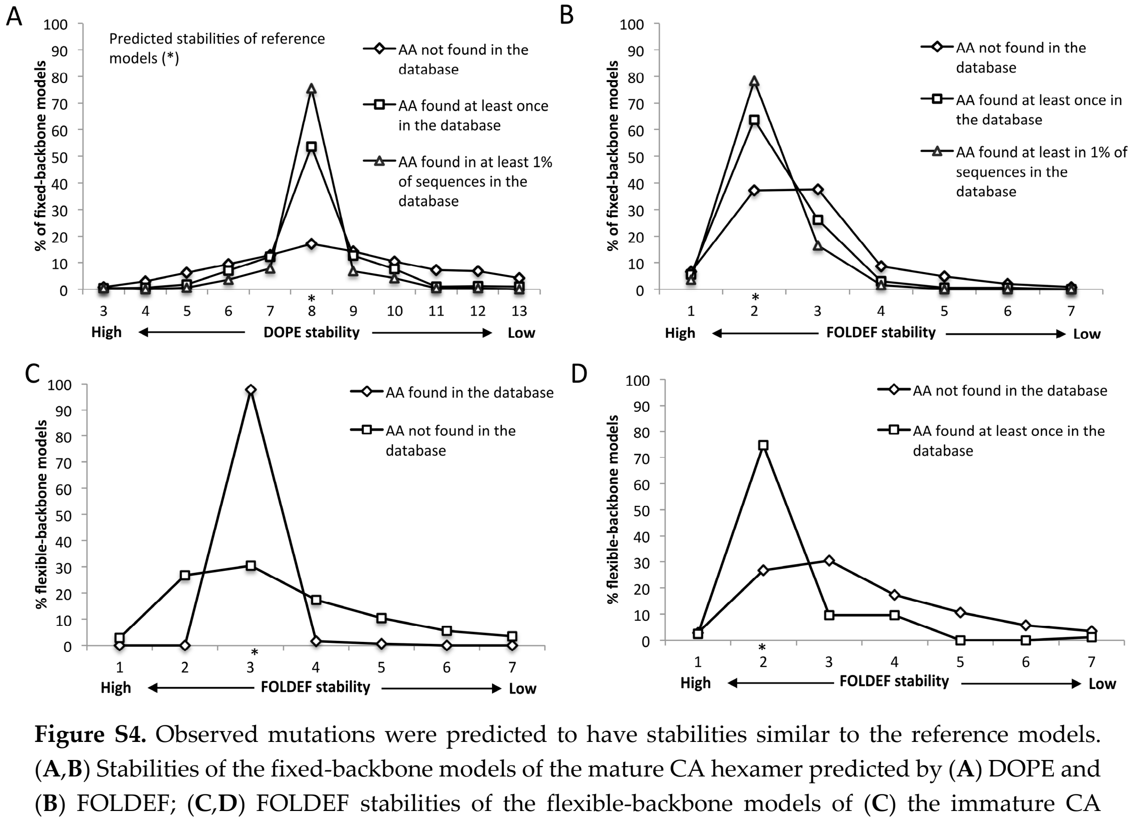

Figure S4. Observed mutations were predicted to have stabilities similar to the reference models. (**A**,**B**) Stabilities of the fixed-backbone models of the mature CA hexamer predicted by (**A**) DOPE and (**B**) FOLDEF; (**C**,**D**) FOLDEF stabilities of the flexible-backbone models of (**C**) the immature CA hexamer and (**D**) the CTD of the HOH. * Indicates stability of reference structures. Only results from five higher, five lower and the reference model bins are shown, as they accounted for more than 98% of all models.

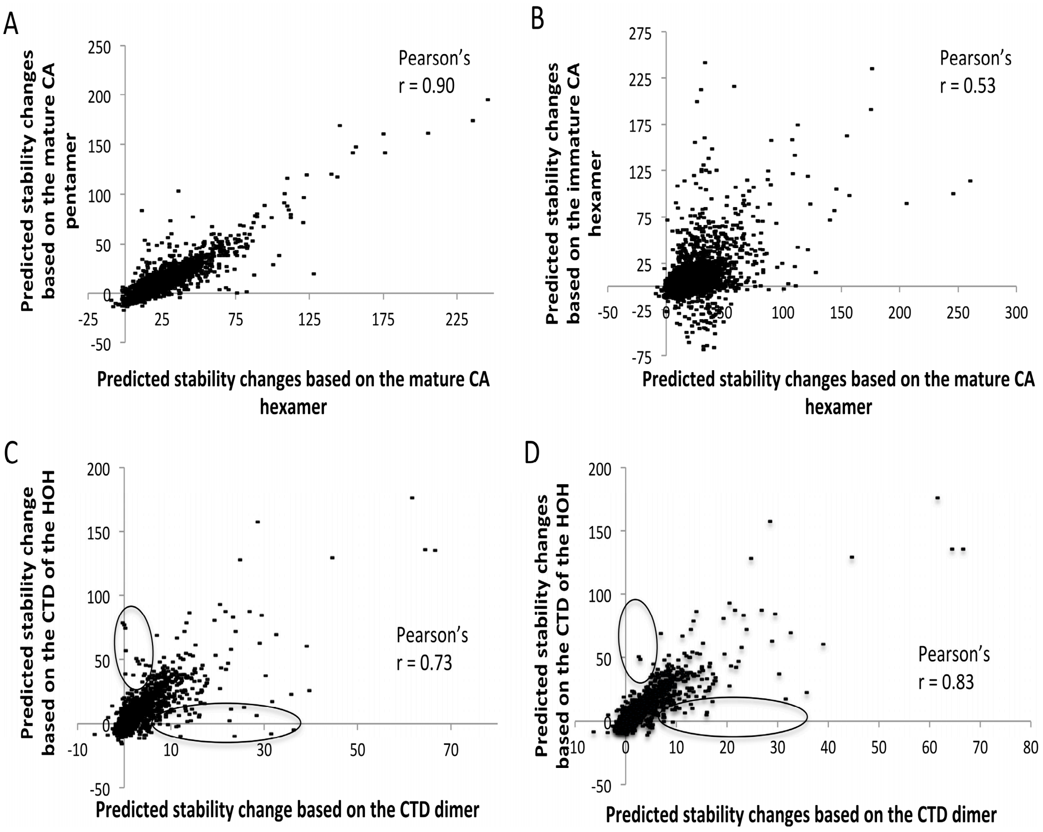

Figure S5. FOLDEF stability correlation between different template. Each dash represents the predicted stability change of each mutant model (**A**) The x- and y-axis values were obtained using the mature CA hexamer and the mature CA pentamer as the template, respectively (**B**) The X-axis values were obtained as in (**A**) while the y-axis values were from the immature CA hexamer; (**C**) The x- and y-axis values were based on the CA-CTD dimer and the CTD of the HOH, respectively; (**D**) same as (**C**) excluding mutations at residues 175, 177, 178, 188, 201, 204, 207 and 208 as highlighted by the two circles.

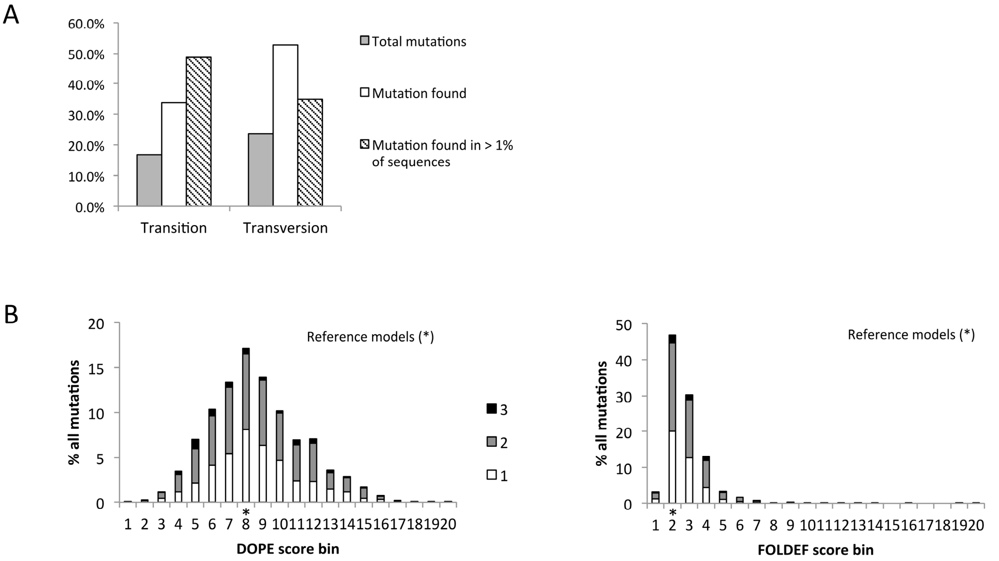

Figure S6. Genetics barrier (A) Division of mutations requiring only single nucleotide change into transitions and transversions; (B) Distribution of genetic barrier across all stability levels. Point mutations in each stability bin were classified based on the least number of nucleotide changes needed. Portion of mutations requiring one nucleotide change is shown in white, two in grey, and three in black, respectively.

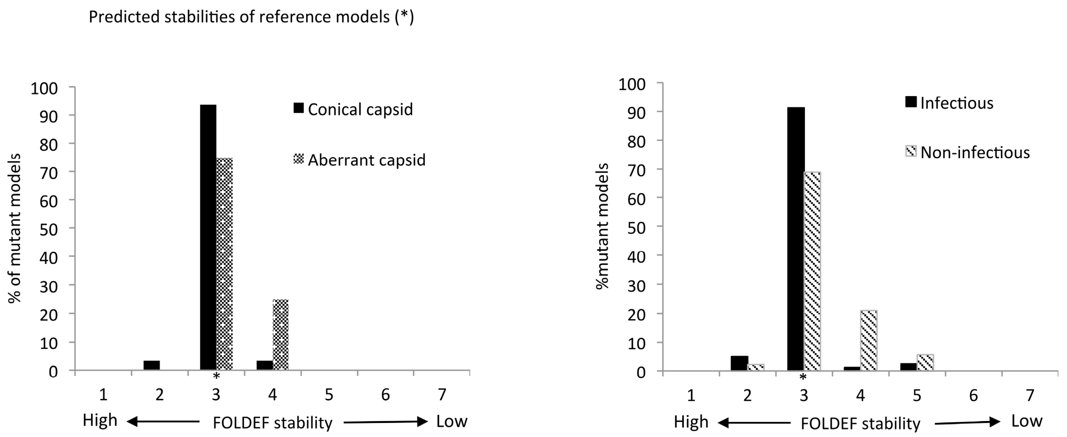

Figure S7. FOLDEF stabilities of mutations with known phenotypes based on flexible-backbone models of the immature CA hexamer.

Table S1. Accuracy of using change in structural stability to predict viral infectivity in binary classification manner.

Template Structure(s)		Accuracy [a]
**NTD**	**CTD**	
Mature CA hexamer (PDB 3H4E)	Mature CA hexamer (PDB 3H4E)	73.68%
Mature CA hexamer (PDB 3H4E)	CTD dimer (PDB 1A43)	75.00%
Mature CA hexamer (PDB 3H4E)	CTD trimer (PDB 3J34)	76.12%
Immature CA hexamer (4USN)	Immature CA hexamer (4USN)	59.28%

[a] Accuracy = (True positive + True negative)/(True positive + True negative + False positive + False negative).

Table S2. Amino acid sites within HIV-1 CA prone to destabilizing mutations.

Site (HXB2)	Consensus AA	Frequency	[a] Solvent Accessibility Surface Area (A^2)	Binding Site of CA Inhibitors
2	ILE	0.98	12.77	None
8	GLY	0.99	84.05	None
20	LEU	0.99	2.1	None
23	TRP	0.99	2.30	CAP-1 [1]; BD3, BM4 [2]; Inhibitor3 [3]
32	PHE	0.99	30.06	CAP-1 [1]; BD3, BM4 [2]; Inhibitor3 [3]; Benzodiazepine series 33 [4]
36	VAL	0.97	1.17	I-XW-053 [5]; BD3 [2]; Inhibitor3 [3]
37	ILE	0.97	23.24	Inhibitor3 [3]
40	PHE	0.99	3.99	Inhibitor3 [3]
43	LEU	0.98	20.56	None
49	PRO	0.99	0	None
52	LEU	0.99	0	None
55	MET	0.99	2.20	None
56	LEU	0.99	11.12	Inhibitor3 [3]
65	ALA	0.99	0	CAP-1 [1]; Inhibitor3 [3]; Benzodiazepine series 33 [4]
66	MET	0.99	5.99	Inhibitor3 [3]; PF-3450074 [6]
69	LEU	0.99	2.69	Inhibitor3 [3]
73	ILE	0.99	7.09	PF-3450074 [6]
80	TRP	0.99	52.06	Inhibitor4 [3]
99	PRO	0.99	14.25	None

Table S2. *Cont.*

Site (HXB2)	Consensus AA	Frequency	[a] Solvent Accessibility Surface Area (A[2])	Binding Site of CA Inhibitors
101	GLY	0.99	2.94	None
104	ILE	0.99	0	None
106	GLY	0.99	8.01	None
109	SER	0.99	14.97	None
111	LEU	0.99	24.66	None
117	TRP	0.99	4.69	Inhibitor4 [3]
126	VAL	0.99	0.29	None
133	TRP	0.99	8.19	None
134	ILE	0.99	1.84	Inhibitor3 [3]
138	LEU	0.98	5.77	Inhibitor3 [3]
141	ILE	0.99	0.18	CAP-1 [1]; Inhibitor3 [3]
142	VAL	0.99	4.90	Inhibitor3 [3]
144	MET	0.99	26.57	None
150	ILE	0.99	3.63	CAC1 [7]; CP4 peptide [8]
153	ILE	0.99	18.47	None
161	PHE	0.99	1.55	None
164	TYR	0.99	0	I-XW-053 [5]; NYAD peptides [9]
165	VAL	0.99	1.65	I-XW-053 [5]; CAI-compound series [10]; NYAD peptides [9]
168	PHE	0.99	0	I-XW-053 [5]; NYAD peptides [9]
169	TYR	0.99	15.6	I-XW-053 [5]; CAI peptide [11]; CAI-compound series [10]; NYAD peptides [9]
172	LEU	0.99	15.58	I-XW-053 [5]; CAC1 [7]; NYAD peptides [9]
189	LEU	0.99	8.37	CAC1 [7]
190	LEU	0.99	5.18	CAC1 [7]
198	CYS	0.99	0	CAC1 [7]
201	ILE	0.99	49.70	CAC1 [7]
202	LEU	0.99	1.28	CAC1 [7]
205	LEU	0.98	60.69	None
206	GLY	0.99	21.01	None
211	LEU	0.99	19.02	CAI-compound series [10]; CAC1 [7]; NYAD peptides [9]
214	MET	0.99	7.30	None
215	MET	0.99	4.80	None

[a] based on the hexamer of hexamer structure (PDB 3J34).

References

1. Kelly, B.N.; Kyere, S.; Kinde, I.; Tang, C.; Howard, B.R.; Robinson, H.; Sundquist, W.I.; Summers, M.F.; Hill, C.P. Structure of the antiviral assembly inhibitor CAP-1 complex with the HIV-1 CA protein. *J. Mol. Biol.* **2007**, *373*, 355–366.
2. Lemke, C.T.; Titolo, S.; von Schwedler, U.; Goudreau, N.; Mercier, J.F.; Wardrop, E.; Faucher, A.M.; Coulombe, R.; Banik, S.S.; Fader, L.; *et al.* Distinct effects of two HIV-1 capsid assembly inhibitor families that bind the same site within the N-terminal domain of the viral CA protein. *J. Virol.* **2012**, *86*, 6643–6655.

3. Goudreau, N.; Coulombe, R.; Faucher, A.M.; Grand-Maitre, C.; Lacoste, J.E.; Lemke, C.T.; Malenfant, E.; Bousquet, Y.; Fader, L.; Simoneau, B.; *et al.* Monitoring binding of HIV-1 capsid assembly inhibitors using (19)F ligand-and (15)N protein-based nmr and X-ray crystallography: Early hit validation of a benzodiazepine series. *Chem. Med. Chem.* **2013**, *8*, 405–414.
4. Tremblay, M.; Bonneau, P.; Bousquet, Y.; DeRoy, P.; Duan, J.; Duplessis, M.; Gagnon, A.; Garneau, M.; Goudreau, N.; Guse, I.; *et al.* Inhibition of HIV-1 capsid assembly: Optimization of the antiviral potency by site selective modifications at N1, C2 and C16 of a 5-(5-furan-2-yl-pyrazol-1-yl)-1h-benzimidazole scaffold. *Bioorg. Med. Chem. Lett.* **2012**, *22*, 7512–7517.
5. Kortagere, S.; Madani, N.; Mankowski, M.K.; Schon, A.; Zentner, I.; Swaminathan, G.; Princiotto, A.; Anthony, K.; Oza, A.; Sierra, L.J.; *et al.* Inhibiting early-stage events in HIV-1 replication by small-molecule targeting of the HIV-1 capsid. *J. Virol* **2012**, *86*, 8472–8481.
6. Blair, W.S.; Pickford, C.; Irving, S.L.; Brown, D.G.; Anderson, M.; Bazin, R.; Cao, J.; Ciaramella, G.; Isaacson, J.; Jackson, L.; *et al.* HIV capsid is a tractable target for small molecule therapeutic intervention. *PLoS Pathog.* **2010**, *6*, e1001220.
7. Bocanegra, R.; Nevot, M.; Domenech, R.; Lopez, I.; Abian, O.; Rodriguez-Huete, A.; Cavasotto, C.N.; Velazquez-Campoy, A.; Gomez, J.; Martinez, M.A.; *et al.* Rationally designed interfacial peptides are efficient *in vitro* inhibitors of HIV-1 capsid assembly with antiviral activity. *PLoS ONE* **2011**, *6*, e23877.
8. Dewan, V.; Liu, T.; Chen, K.M.; Qian, Z.; Xiao, Y.; Kleiman, L.; Mahasenan, K.V.; Li, C.; Matsuo, H.; Pei, D.; *et al.* Cyclic peptide inhibitors of HIV-1 capsid-human lysyl-tRNA synthetase interaction. *ACS Chem. Biol.* **2012**, *7*, 761–769.
9. Zhang, H.; Curreli, F.; Zhang, X.; Bhattacharya, S.; Waheed, A.A.; Cooper, A.; Cowburn, D.; Freed, E.O.; Debnath, A.K. Antiviral activity of α-helical stapled peptides designed from the HIV-1 capsid dimerization domain. *Retrovirology* **2011**, *8*, 28.
10. Curreli, F.; Zhang, H.; Zhang, X.; Pyatkin, I.; Victor, Z.; Altieri, A.; Debnath, A.K. Virtual screening based identification of novel small-molecule inhibitors targeted to the HIV-1 capsid. *Bioorg Med. Chem* **2011**, *19*, 77–90.
11. Ternois, F.; Sticht, J.; Duquerroy, S.; Krausslich, H.G.; Rey, F.A. The HIV-1 capsid protein C-terminal domain in complex with a virus assembly inhibitor. *Nat. Struct Mol. Biol* **2005**, *12*, 678–682.
